# Doxycycline vs azithromycin in patients with scrub typhus: a systematic review of literature and meta-analysis

**DOI:** 10.1186/s12879-023-08893-7

**Published:** 2023-12-18

**Authors:** Nitin Gupta, Carl Boodman, Christelle Genevieve Jouego, Steven Van Den Broucke

**Affiliations:** 1 Department of Infectious Diseases, Kasturba Medical College, Manipal, Manipal Academy of Higher Education, Manipal, 576104 India; 2 Department of Clinical Sciences, Institute of Tropical Medicine, Nationalestraat 155, 2000 Antwerp, Belgium; 3 Division of Infectious Diseases, Department of Internal Medicine, University of Manitoba, Winnipeg, MB Canada; 4 Molecular Diagnostic and Research Group, University of Yaoundé, 11864 Yaoundé, Cameroon

**Keywords:** Orientia tsutsugamushi, Fever, Defervescence, Mortality

## Abstract

**Introduction:**

Scrub typhus is a bacterial mite-borne disease associated with poor clinical outcomes if not treated adequately. The study aimed to compare the time to defervescence, clinical failure, mortality and treatment-related adverse effects of two common drugs (doxycycline and azithromycin) used for its treatment.

**Methodology:**

This was a systematic review and meta-analysis. All studies up to 20.03.2023 were screened for eligibility in Pubmed and Embase using a search string containing terms related to scrub typhus, doxycycline and azithromycin. After two phases of screening, all comparative studies where doxycycline and azithromycin were used to treat scrub typhus were included. The studies were critically appraised using standardised tools, and a meta-analysis was performed for time to defervescence (primary outcome), clinical failure, mortality and treatment-related adverse effects.

**Results:**

Of 744 articles from two databases, ten were included in the meta-analysis. All but two studies had a high risk of bias. The meta-analysis for time to defervescence had a high heterogeneity and did not show any significant difference between doxycycline and azithromycin arms [Mean difference of -3.37 hours (95%CI: -10.31 to 3.57), *p*=0.34]. When the analysis was restricted to studies that included only severe scrub typhus, doxycycline was found to have a shorter time to defervescence [mean difference of -10.15 (95%CI: -19.83 to -0.46) hours, *p*=0.04]. Additionally, there was no difference between the two arms concerning clinical failure, mortality and treatment-related adverse effects.

**Conclusion:**

The current data from studies with a high risk of bias did not find statistically significant differences in clinical outcomes between doxycycline and azithromycin for scrub typhus

**Supplementary Information:**

The online version contains supplementary material available at 10.1186/s12879-023-08893-7.

## Introduction

Acute undifferentiated febrile illness (AUFI) is a broad syndromic term that includes infections with a fever of short duration without specific localisation [1]. It is a common cause of morbidity and mortality in tropical countries [1]. Scrub typhus, a mite-borne infection caused by *Orientia tsutsugamushi*, is a leading cause of AUFI in Asia [2]. The disease was initially thought to be restricted to an imaginary triangle called the ‘tsutsugamushi triangle’ (Indian subcontinent, South-East Asian countries, China, Korea, Japan, and Northern Australia) [3]. However, recent cases have also been reported in Peru and Kenya [3]. Despite the high burden, scrub typhus is neglected, with limited high-quality studies available to inform treatment decisions [4]. In a systematic review from India, scrub typhus was the aetiology in a quarter of hospitalised patients with AUFI [5]. Around 20% of scrub typhus patients had severe manifestations and required intensive care [5]. The median mortality of scrub typhus is 6% in untreated patients, but it falls to 1.4% in adequately treated patients [2]. In a systematic review of the treatment landscape, doxycycline and azithromycin were the most common antibiotics used for management [4]. Indian Guidelines recommend either drug to treat scrub typhus without any clear preference [6]. With increasing awareness and improved diagnostics, many comparative studies on the management of scrub typhus have recently been published. There is a need for a meta-analysis combining the results of all published studies, including the recent ones, to identify the drug with better clinical outcomes. This study aimed to compare time to defervescence, clinical failure, mortality and treatment-related adverse effects between doxycycline and azithromycin in patients of all ages and sexes diagnosed with scrub typhus.

## Methodology

### Search strategy

This systematic review and meta-analysis was conducted after Prospero registration (CRD42023409680). The search string was developed using relevant search terms for population (febrile patients diagnosed with scrub typhus) and intervention/comparator (doxycycline and azithromycin) (Supplementary Table 1).

### Study selection

Articles in all languages from the two databases (Pubmed and Embase) up to 20/03/2023 were included. The detailed eligibility criteria have been tabulated (Supplementary Table 2). Briefly, randomised controlled trials (RCTs) and non-randomized clinical trials (nRCTs) on scrub typhus, where doxycycline and azithromycin were used for the treatment of individuals of any age and sex (including pregnant individuals) diagnosed with scrub typhus were included. Studies on non-human subjects and those focusing exclusively on coinfections were excluded. Case reports, case series, reviews, systematic reviews, conference abstracts and letters to the editor were also excluded.

The articles were retrieved from the two databases after removing the duplicates. Two authors (NG and CB) independently screened the titles and abstracts for eligibility. After the initial screening, full-length articles were retrieved for screening by the same two authors and the articles that met the eligibility criteria were included in the final analysis. The third author (SVDB) was consulted for conflicts between the two reviewers.

### Outcome measures

The primary outcome measure of the study was time to defervescence. The secondary outcome measures were clinical failure, mortality and treatment-related adverse effects. Time to defervescence was defined as the time taken for the temperature to go below a cut-off defervescent point. This was only considered if the fall in temperature was sustained for more than 24 hours. Clinical failure was defined as the proportion of patients not attaining defervescence in two or three days after initiation of treatment. The all-cause mortality during the follow-up period in each treatment arm was also noted. In those studies where all-cause mortality was unavailable, scrub-typhus-related mortality was recorded. Furthermore, adverse effects in each arm attributed to either doxycycline or azithromycin were recorded.

### Data extraction

The following population-related details were extracted from studies: author details, type of study, diagnostic modality, age group of the included patients (adult or paediatric) and disease severity. Severe scrub typhus was defined as the presence of any organ dysfunction. The number of patients with severe scrub typhus was recorded in each study. The dose, duration, and route of doxycycline and azithromycin were recorded. The definition of fever and how the temperature was measured were recorded for each study. The mean or median time to defervescence of fever in hours, proportion of patients with clinical failure, proportion of patients who died and proportion of patients with treatment-related adverse effects in each treatment arm were noted.

### Critical appraisal of literature

Each included article was appraised using the Cochrane Risk of Bias tool for RCTs and the ROBINS-1 tool for nRCTs (including analytical observational studies) [7, 8].

### Data analysis

Results from all the studies were compared to determine differences in the outcome measures between doxycycline and azithromycin. The mean difference of time to defervescence was pooled using a random-effect model (inverse variance method), and the results were represented by a point estimate with a 95% confidence interval. In the studies where mean time to defervescence and standard deviation were not mentioned, they were indirectly calculated from the median and interquartile range (or range) using the method suggested by Wan et al. [9]. The meta-analysis results were stratified according to the study type, age groups and severity. The risk ratio for clinical failure, mortality and adverse events was pooled using a random-effect model (Mantel Haenszel method), and the results were represented by a point estimate with a 95% confidence interval. The heterogeneity across studies for all the outcomes was tested using Tau^2^, Chi^2^ and *I*^2^ tests. An I^2^ of more than 60% was taken as significant heterogeneity. Review Manager (version 5.3, Cochrane Nordic, Copenhagen, Denmark) was used for the meta-analysis.

## Results

### Study selection

**Fig. 1:**
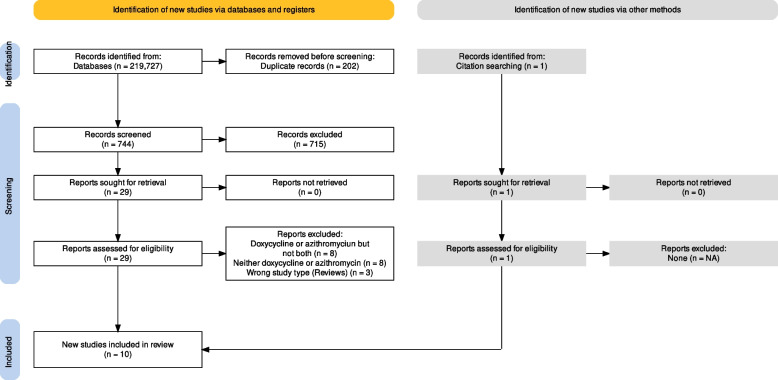
PRISMA diagram showing the two screening phases and the number of included and excluded articles

A total of 219 articles were retrieved from Pubmed, and 727 were retrieved from Embase. After duplicate deletion, a total of 744 articles were included. After the title abstract screening, 29 articles were included for full-text screening. Nineteen papers were excluded during the full-text review. Ten articles were finally included for data extraction and analysis (Fig. 1).

### Single-arm studies (not included)

Eight studies had data on time to defervescence for either azithromycin or doxycycline but not both [10–17]. They were, therefore, not included in the final meta-analysis. The average time to defervescence in these studies ranged from 24 to 52 hours for doxycycline and 24 to 43 hours for azithromycin (Supplementary Table 3).

### Baseline characteristics of included studies

Ten studies with 2856 patients were included in this analysis, with sample sizes ranging from 57 to 780 [18–27] (Table 1). Seven of the ten studies were published during or after 2021. Most studies were reported from India (*n*=5, 50%). There were five RCTs [18, 21, 25–27]. Four studies included only paediatric patients (less than 14-18 years) [21, 22, 24, 25]. The remaining six incorporated either adults or individuals of any age. Scrub typhus was defined primarily based on the presence of eschar and/or serological tests (Weil Felix test or, rapid diagnostic tests or Enzyme-linked immunosorbent assay or Immunofluorescence assay). Additional details on the diagnostic tests used for diagnosis have been summarised in Supplementary Table 4. Six studies excluded the use of antibiotics (active for scrub typhus) before presentation (Supplementary Table 4). Only one study explicitly mentioned that co-infections were ruled out [24]. Four studies exclusively focussed on patients with mild scrub typhus [21, 25–27]. Three studies focused entirely on severe scrub typhus patients [18, 19, 23]. The other three studies had severe scrub typhus ranging from 16% to 51% [20, 22, 24].

**Table 1:** Comparative studies for time to defervescence or mortality on doxycycline vs. azithromycin for scrub typhus

Sn	Author	Year of Study	Country	Type of Study	Age group	Eschar	Serology	Sample size	Severe scrub typhus
1	Varghese 2023 [18]	2018-2022	India	Randomised controlled trial	Adult (>15 years)	Yes	RDT	528	528 (100%)
2	Guan-Xiu-Gang 2022 [19]	2012-2018	China	Retrospective cohort study	Adult and Paediatric	NA	Weil Felix test or IFA	780	780 (100%)
3	Hwang 2022 [20]	2013-2021	South Korea	Retrospective cohort study	Adult (>18 years)	NA	IFA	326	165 (51%)
4	Kabir 2022 [21]	2019-2021	India	Randomised controlled trial	Paediatric (<15 years)	Yes	Weil Felix test or ELISA	114	0
5	Arun Babu 2021 [22]	2015-2019	India	Prospective cohort study	Paediatric (<12 years)	NA	ELISA	660	203 (31%)
6	Barnabas 2021 [23]	2013-2015	India	Prospective cohort study	Adult (≥15 years)	NA	ELISA	103	103 (100%)
7	Veerappan 2021 [24]	2017-2020	India	Retrospective cohort study	Paediatric (<14 years)	NA	Rapid test	138	22 (16%)
8	Chanta 2015 [25]	2010-2013	Thailand	Randomised controlled trial	Paediatric (<15 years)	Yes	Rapid test	57	0
9	Phimda 2007 [26]	2003-2005	Thailand	Randomised controlled trial	Adult (>14 years)	NA	IFA	57	0
10	Kim 2004 [27]	2002-2003	South Korea	Randomised controlled trial	Adult (>18 years)	NA	IFA	93	0

*Abbreviations*: *IFA* Immunofluorescence assay, *ELISA* Enzyme-linked immunosorbent assay, *NA* No information available

### Outcomes listed in the included studies

All the included studies had time to defervescence as either a primary (*n*=3) or a secondary outcome (*n*=7) (Supplementary Table 5). Clinical failure (as defined in the methodology) was reported in nine studies. It was reported as the primary outcome in five studies. Mortality was mentioned in six studies [18–20, 23, 26, 27]. Only one of the studies mentioned mortality as the primary outcome. Adverse drug reactions were reported in five studies.

### Definitions of outcomes

The definition of time to defervescence differed across the studies. The period of sustained temperature fall below a pre-defined cut-off (measured by oral or axillary thermometer) required to define defervescence varied from 24 to 72 hours across studies (Table 2). Only five out of ten studies mentioned that time to defervescence was calculated after explicitly excluding the use of antipyretics. Only five studies mentioned the frequency of temperature measurement to see when the temperature fell below the cut-off point. Except for one study, which monitored temperature 24 hourly, the rest of the four studies measured temperature every two to four hours (Table 2). Of the nine studies that reported clinical failure, seven reported failure to defervesce at two days. The two remaining studies reported failure to defervesce at three days (Table 2). Four studies defined mortality in their methodology. Two studies reported scrub-typhus-related mortality, while the other two reported all-cause mortality [18–20, 23]. Two additional studies reported mortality but did not define them [26, 27].

**Table 2:** Definition of outcomes for the studies included in the meta-analysis

Author	Time to defervescence					Clinical failure	Mortality	Adverse drug effect
	Below Temp (^o^C)	Sustained (hrs)	Antipyretics excluded	Thermometer	Frequency	Cut-off day for defervescence		
Varghese 2023 [18]	37.5	24	NA	Oral	q24h	2 days^a^	All-cause	Treatment-related ≥ Grade 3
Guan-Xiu-Gang 2022 [19]	37.3	48	NA	NA	NA	NR	ST related	NA
Hwang 2022 [20]	37.3	48	Yes	NA	NA	2 days	ST related	NA
Kabir 2022 [21]	37.3	48	NA	axillary	Q4h	3 days	NA	Any treatment related
Arun Babu 2021 [22]	37.5	24	NA	NA	NA	2 days	NA	NA
Barnabas 2021 [23]	37.7	72	Yes	axillary	NA	2 days	All-cause	NA
Veerappan 2021 [24]	37.2	48	Yes	axillary	Q2h	2 days	NA	NA
Chanta 2015 [25]	37.3	48	NA	NA	NA	3 days	NA	Any treatment related
Phimda 2007 [26]	37.5	NA	Yes	oral	q4h	2 days^a^	NA	Any treatment related
Kim 2004 [27]	37.3	48	Yes	oral	q2h	2 days^a^	NA	Reported

*Abbreviations* *NA* Data Not Available, *ST* Scrub typhus, *q24h* Every 24 hours, *q4h* Every 4 hours, *q2h* Every 2 hours

^a^Three studies primarily used 5 days as the cut-off for defervescence to define clinical failure. Data on the percentage failing to defervesce at 2 days was extracted for this review from these studies

### Details of the interventions

Doxycycline and azithromycin were used at similar dosages. Doxycycline in adult studies was used at 100 mg twice daily, whereas in paediatric patients, it was given at 2.2 mg/kg twice daily. Similarly, azithromycin was dosed at 500 mg (in adults) or 10 mg/kg (in children) once daily. Loading dose was given in three studies (Table 3). Most studies used oral formulations of both drugs except for the two studies on severe scrub typhus, where intravenous formulations were used. Additionally, azithromycin was used in intravenous formulation in the study by Hwang et al. The duration of treatment for doxycycline ranged from 5 to 8 days, while the course of treatment for azithromycin ranged from 1 to 7 days (Table 3). The most common duration for both drugs was seven days.

**Table 3:** Outcomes according to intervention in studies included in the meta-analysis

Author	Drug	Dosage	Duration (days)	Number of cases	Average TOD (hours)	Clinical failure (2-3 days)	Clinical failure (5 days)	Mortality (%)	Adverse drug effect
Varghese 2023 [18]	IV Doxy	200 mg BD on D1 f/b 100 mg BD	7	265	96*	33/254 (13%)	8/243 (3%)	29 (10.9%)	0^b^
	IV Azithro	500 mg BD on D1 f/b 500 mg OD	7	263	96*	35/251 (14%)	16/225 (7%)	32 (12.2%)	0^b^
Guan-Xiu-Gang 2022 [19]	IV Doxy	100 mg BD	7	701	48*	NA	NA	30 (4.3%)	NA
	IV Azithro	500 mg OD	6	79	48*	NA	NA	4 (5.1%)	NA
Hwang 2022 [20]	Oral Doxy	100 mg BD	8	217	72*	66/108 (61%)	NA	0 (0%)	NA
	IV Azithro	500 mg OD	4	109	72*	64/109 (59%)	NA	1 (0.9%)	NA
Kabir 2022 [21]	Oral Doxy	2.2 mg/kg BD	5	58	25.8	2 (4%)	NA	NA	5 (9%)
	Oral Azithro	10 mg/kg OD	5	56	24.5	1 (2%)	NA	NA	1 (2%)
Arun Babu 2021 [22]	Oral Doxy	2.2 mg/kg BD	7	344	44.6	49 (14%)	NA	NA	NA
	Oral Azithro	10 mg/kg OD	7	316	49.9	76 (24%)	NA	NA	NA
Barnabas 2021 [23]	Oral Doxy	NA	7	46	30.5	11 (24%)	NA	1 (2.2%)	NA
	Oral Azithro	NA	7	57	38.5	20 (35%)	NA	6 (10.5%)	NA
Veerappan 2021 [24]	Oral Doxy	2.2 mg/kg BD	NA	76	12*	6 (8%)	NA	NA	NA
	Oral Azithro	10 mg/kg OD	NA	62	24*	16 (26%)	NA	NA	NA
Chanta 2015 [25]	Oral Doxy or chloramphenicol	d-2.2 mg /kg BD, c-25mg/kg QID	5	28 (d-9, c-19)	30*	4(14%)	NA	NA	0 (0%)
	Oral Azithro	20 mg/kg on D1 f/b 10 mg/kg	3	29	36*	5 (17%)	NA	NA	1 (3%)
Phimda 2007 [26]	Oral Doxy	200 mg stat f/b 100 mg BD	7	27	48*	11 (41%)	0 (0%)	0 (0%)	27.6%^a^
	Oral Azithro	1g stat f/b 500 mg OD	3	30	60*	21 (70%)	1 (3%)	0 (0%)	10.6%^a^
Kim 2004 [27]	Oral Doxy	100 mg BD	7	46	29*	13 (28%)	3 (7%)	0 (0%)	16 (35%)
	Oral Azithro	500 mg stat	1	47	21*	7 (15%)	0 (0%)	0 (0%)	13 (28%)

*Abbreviation*: *Doxy or d* Doxycycline, *azithro* Azithromycin, *c* Chloramphenicol, *mg* Milligrams, *kg* kilograms, *D1* day 1, *BD* Twice daily, *OD* Once daily, *f/b* Followed by, *iv* Intravenous, *TOD* Mean time to defervescence (*Median), *NA* Data Not Available

^a^Adverse events were reported in patients receiving doxycycline and azithromycin for scrub typhus or leptospirosis patients. Separate data on scrub typhus was not available

^b^Grade 3 or higher treatment-related adverse events

### Effect estimates in the individual studies

The time to defervescence with doxycycline ranged from 12 to 96 hours, while it was 21 to 96 hours for azithromycin (Table 3). The clinical failure rates (Day 2-3) varied from 4-41% in doxycycline and 2-70% in azithromycin (Table 3). When the cut-off for attaining defervescence was taken as five days to define failure, the failure rates in both arms varied between 0 and 7%. Mortality with doxycycline and azithromycin ranged from 0 to 10.9% and 0 to 12.2%, respectively (Table 3).

### Critical appraisal of the included studies

**Fig. 2:**
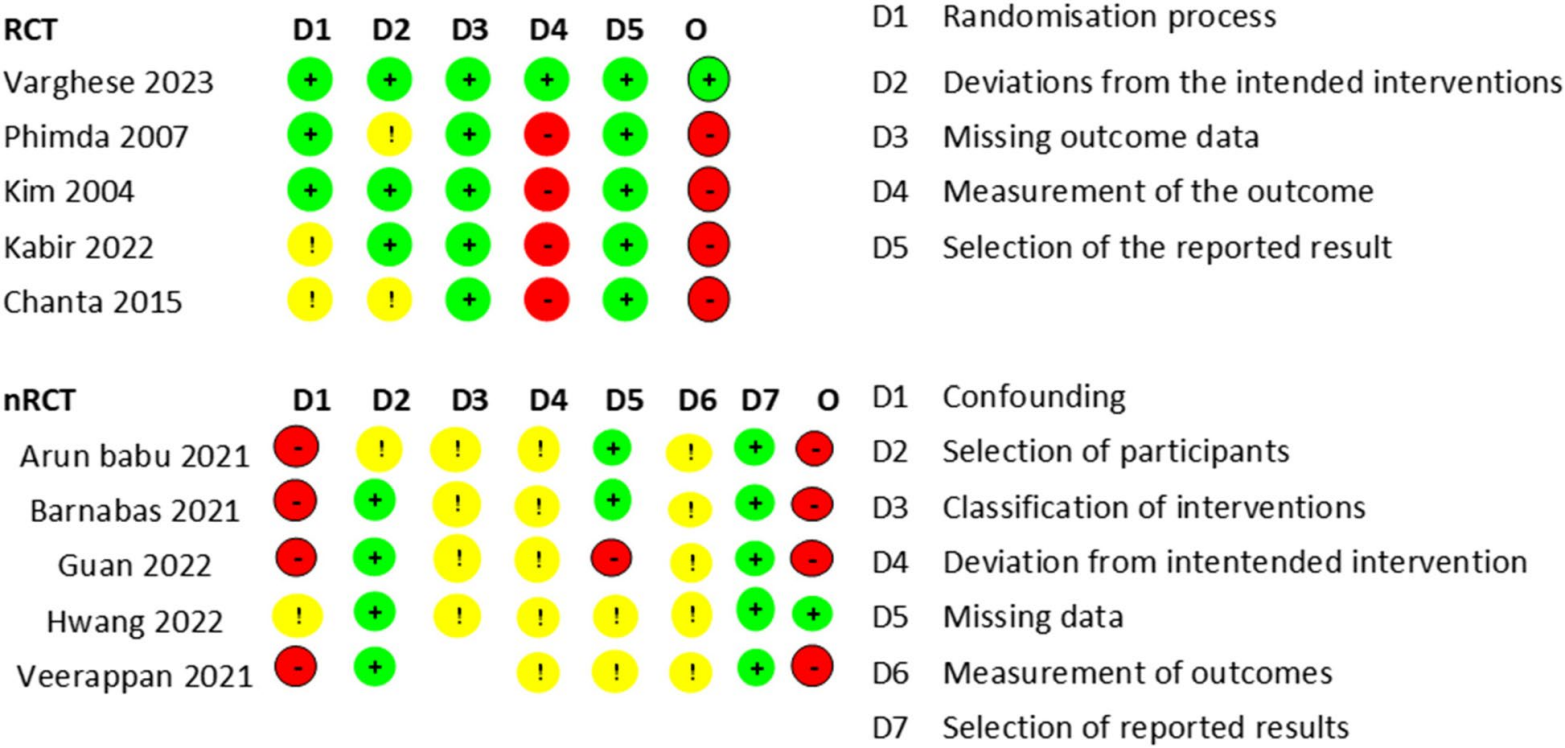
Risk of bias analysis using (**A**) Cochrane Risk of Bias tool for randomised controlled trials and (**B**) ROBINS-1 tool for non-randomised clinical trials (including observational studies). Abbreviation: RCT- Randomised controlled trials, nRCTs- non-Randomised controlled trials, D- Domains of bias. *Colour grading- Red- High risk of bias, Yellow- Moderate risk of bias and Green- Low risk of bias. D1 to D7 stands for the various domains of critical appraisal

The critical appraisal of the five RCTs showed that only one had a low risk of bias overall. The rest of the four had a high risk of bias (Fig. 2). The critical appraisal of the n-RCTs showed that all of them had a moderate or high risk of bias (Fig. 2).

### Time to defervescence meta-analysis

**Fig. 3:**
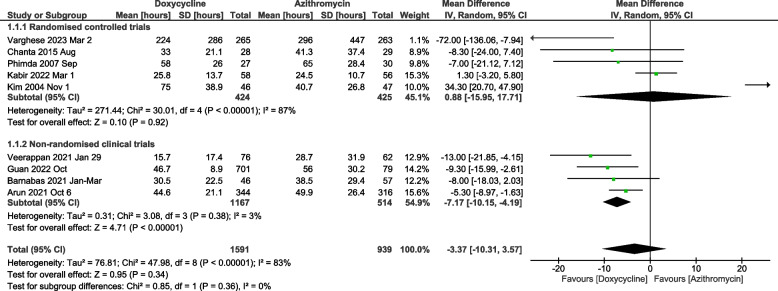
Meta-analysis of doxycycline vs. azithromycin for mean time to defervescence classified according to study type

**Fig. 4:**
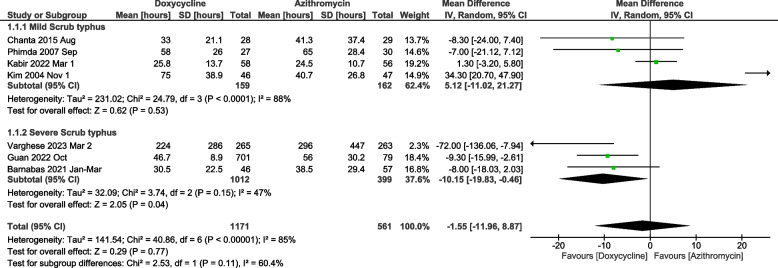
Meta-analysis for time to defervescence with studies stratified according to severity

Nine studies were included in the meta-analysis for the time to defervescence. The study by Hwang et al. could not be included in the time to defervescence meta-analysis as it just reported the median without reporting the standard deviation, range or inter-quartile range [20]. The meta-analysis of mean time to defervescence between doxycycline and azithromycin showed very high heterogeneity and did not show any difference [Mean difference of -3.37 hours (95%CI: -10.31 to 3.57), *p*=0.34] (Fig. 3). There was no difference between the two arms when the analysis was restricted to randomised controlled trials (Fig. 3). No significant differences in the adult and paediatric subgroups were noticed concerning the mean time to defervescence (Supplementary Fig. 1). An additional sensitivity analysis was done where the studies that focussed exclusively on mild or severe scrub typhus were included. The meta-analysis of studies which included severe patients showed that the mean time to defervescence with doxycycline was significantly lower when compared to azithromycin [mean difference of -10.15 (95%CI: -19.83 to -0.46) hours, *p*=0.04] (Fig. 4). The I^2^ for heterogeneity was 47%. Similar results were obtained when the standardised mean difference was taken as the effect measure of choice (Supplementary Fig. 2).

A separate subgroup analysis was conducted between studies that used loading dose and studies that did not. Although heterogeneity was decreased in those who received loading doses, no significant difference between doxycycline and azithromycin was noted in the two subgroups (Supplementary Fig. 3).

### Clinical failure meta-analysis

**Fig. 5:**
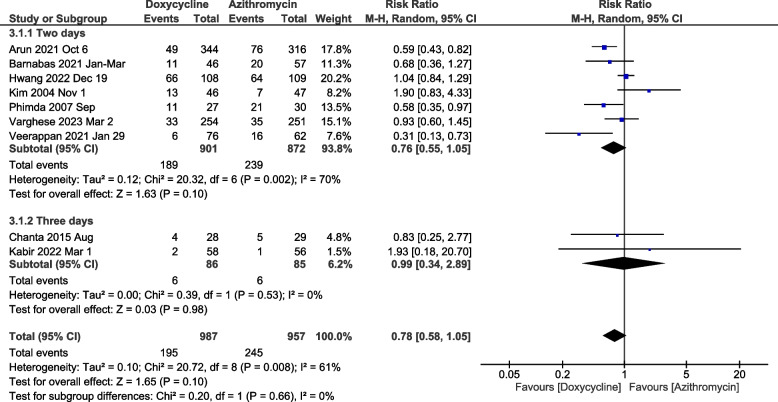
Meta-analysis of doxycycline vs. azithromycin showing clinical failure rates on Days 2 to 3

The meta-analysis comparing clinical failure rates (Days 2-3) in nine included studies did not show any difference between the two arms [RR-0.78 (95%CI: 0.58-1.05), *p*=0.1] (Fig. 5). On sub-group analysis, the doxycycline arm showed a lesser risk for clinical failure on day two, but the difference was not significant [0.76 (95%CI: 0.55-1.05), *p*=0.1]. When failure to defervesce was evaluated on Day 5, the difference in clinical failure rates was insignificant between both arms [RR- 0.78 (95%CI: 0.16-3.69), *p*=0.75] (Supplementary Fig. 4).

### Mortality meta-analysis

**Fig. 6:**
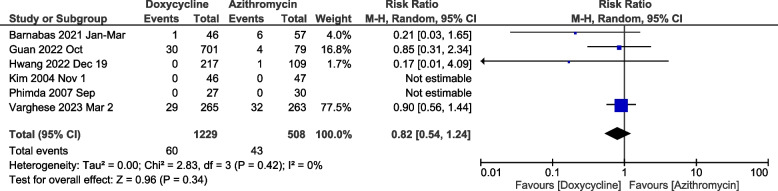
Meta-analysis of doxycycline vs. azithromycin for mortality

Two of the six studies reported no mortality in either arm. Since the meta-analysis of dichotomous variables does not consider studies with no outcome events, only four studies were used for mortality meta-analysis [26, 27]. Mortality in the two arms was not significantly different [RR: 0.82 (95%CI: 0.54-1.24), *p*=0.34] (Fig. 6). There was no significant heterogeneity when studies were evaluated to calculate the risk ratio for mortality.

### Treatment-related adverse effects meta-analysis

Of the five studies that reported treatment-related adverse effects, one reported only those adverse effects that were Grade 3 or higher [18]. In the study by Phimda et al., adverse effects of patients diagnosed with either scrub typhus or leptospirosis were reported without making any distinction between them [26]. Therefore, only three studies were included in the meta-analysis. Both arms had similar treatment-related adverse effects [RR-1.37 (95%CI: 0.64-2.94). *p*=0.33] (Supplementary Fig. 5).

Because fewer than ten studies were in the meta-analysis, funnel plots were not created for any outcomes.

## Discussion

*Orientia tsutsugamushi* is a gram-negative organism belonging to the Rickettsiaceae family [28]. Owing to its intracellular nature, scrub typhus can only be treated by antibiotics that accumulate in large quantities within the target cells. The first antibiotic used to treat scrub typhus was chloramphenicol [28]. Due to its significant toxicity profile, it has been predominantly replaced by doxycycline and azithromycin [18]. In this systematic review and meta-analysis, after screening a total of 744 articles published before March 2023, 10 articles were included in the final meta-analysis. This study included seven additional comparative articles not included in the previous meta-analyses [28, 29]. There was no significant difference between doxycycline and azithromycin concerning the time to defervescence, clinical failure, mortality and treatment-related adverse effects.

While both drugs act by inhibiting protein synthesis, there are significant differences in their mechanism of action [29]. Doxycycline acts on the 30S ribosomal unit and inhibits protein synthesis, which might lead to a quicker reduction in the bacterial load. However, azithromycin, acting on the 50S ribosomal unit, may have a longer-lasting effect on bacterial replication, potentially explaining the comparable clinical outcomes in terms of time to defervescence [29]. The two antibiotics also differ according to the WHO Access Watch and Reserve (AWARE) antibiotic classification [30]. While doxycycline is classified as an ‘Access’ group antibiotic, azithromycin is classified into the ‘Watch’ group [31]. This also has a bearing on the cost and availability of these drugs.

Time to defervescence was selected as the primary outcome in this SR as it was amongst the commonest primary outcomes in the selected studies. A longer time to defervescence leads to unnecessary escalation of antibiotics. Since fever is the most important presenting feature of scrub typhus, clinicians keep adding additional antibiotics until defervescence in routine clinical practice. In a randomised controlled trial from India, the average number of antimicrobials prescribed to patients with AUFI was 2.5 [32]. Overuse of antimicrobials can fuel antimicrobial resistance, and since scrub typhus endemic areas are also faced with significant antimicrobial resistance, choosing an intervention that decreases antimicrobial overuse is pertinent. Also, increased time to defervescence is associated with more extended hospital stays, thereby increasing the hospital burden and risk of acquisition of nosocomial infections. We chose to analyse time-to-defervescence as a continuous outcome (mean difference) and not a time-to-event analysis, as almost all study participants who contributed some period of time ended in the event (defervescence). Besides, the log hazard ratio required for conducting a time-to-event meta-analysis was unavailable in all the studies.

Nine studies with moderate to high risk of bias used in meta-analysis of time to defervescence revealed significant heterogeneity and no significant difference between the two arms, even when the analysis was restricted to RCTs. Since observational studies tend to exaggerate the true effect estimates, we did not attribute much importance to the sub-group analysis of nRCTs showing shorter defervescence with doxycycline. To reduce the heterogeneity in the primary outcome measure, we conducted a sensitivity analysis of studies that included only severe scrub typhus. The heterogeneity decreased substantially in this sensitivity analysis; doxycycline performed better than azithromycin.

All except one RCT by Varghese et al. had a high risk of bias in the quality assessment [18]. Despite the low risk of bias in the trial, the temperature measurement strategy (24 hourly) was not ideal for detecting differences of less than 24 hours between the two arms. The study also reported a wide interquartile range in both arms (4 to 20 days in doxycycline and 4 to 28 days in the azithromycin arms). It is unusual to see fever persisting for up to 28 days in scrub typhus. It is possible that these critically ill patients might have had other causes of fever (thrombophlebitis, hospital-acquired infection, etc) after the actual defervescence was obtained. The indirectly calculated mean and standard deviation in Varghese et al. ranged from 224 to 296 hours and 286 to 447 hours, respectively. The mean in other studies ranged from 24 to 65 hours, while the standard deviation ranged from 10 to 39 hours. Despite a substantial sample size, the weight of the study in this meta-analysis by inverse variance approach was meagre due to unusually high variance. As an additional analysis, we calculated a pooled standardised mean difference to counter the significant variance seen in Varghese’s study (Supplementary Fig. 2). In this analysis, the weight of the Varghese study is significantly higher (16.3%) as compared to the meta-analysis calculating mean-difference (2.3%) (Supplementary Fig. 2). Despite the increased weight, the overall results remain unchanged. Doxycycline has a slightly faster time to defervescence in patients with severe disease but not in mild patients. A standardised mean difference of 0.41 in patients with severe disease would indicate a small to moderate effect size.

In the study by Varghese et al., the combination regimen of doxycycline and azithromycin combination regimen showed better composite outcomes (28-day mortality, persistent complications at Day 7 and persistent fever at Day 5) when compared to doxycycline or azithromycin alone [18]. It must be noted that the combination regimen did not show any significant difference when mortality or persistence of fever were evaluated separately. Lesser persistent complications possibly drove the favourable outcomes in the combination arm. These included persistently high bilirubin (*≥*2mg/dl) or creatinine (≥2mg/dl) at day 7. It can be argued that these are reversible biochemical changes with limited impact on clinically relevant outcomes. Combination therapy was not included as one of the interventions in this meta-analysis due to a lack of similar RCTs focussing on its use.

Traditionally, azithromycin is considered unsafe in children (below eight years of age), as doxycycline use was deemed unsafe because of the problems with dental staining [29, 30]. Recent evidence, however, suggests that doxycycline can be used for short courses in these populations [29]. Four comparative studies in the paediatric population were reported in our meta-analysis. No difference between the two drugs was noted in the paediatric subpopulation. It is also essential to discuss scrub typhus in pregnancy. Of the 33 pregnant patients with scrub typhus in a study from India, poor foetal outcomes (intra-uterine deaths, spontaneous abortion) were observed in more than half of the patients [33]. All patients in this study were treated with azithromycin. Traditionally, azithromycin is considered the safer choice, and as a result, comparative studies between doxycycline and azithromycin in this subgroup have not been reported. With increasing evidence on the safety of doxycycline in pregnancy, it is anticipated that comparative studies between doxycycline and azithromycin will be available.

Clinical failure in most studies was defined as the failure to defervesce in a pre-specified amount of time. Most studies used two or three days as the cut-off. Three studies reported a cut-off at five days and the frequency of patients failing to defervesce at two days. For homogeneity, the cut-off for clinical failure was taken as two or three days. Some studies, such as the one by Varghese et al., used a composite outcome (all-cause mortality at day 28, persistent complications at day 7 and persistent fever at day 5), with one of the outcomes being failure to defervesce [18]. Since the definition of composite outcomes varied across studies, we focussed on these individual outcomes. There was no difference in clinical failure rates between the two drugs. These results of doxycycline achieving somewhat faster defervescence on Day 2 but not Day 3 or 5 can be partially explained by the longer time azithromycin takes to reach the steady state [34].

The meta-analysis for mortality showed no significant difference between doxycycline and azithromycin. This finding is consistent with previous studies and suggests that both antibiotics are equally effective in preventing fatal outcomes in scrub typhus patients. It must be noted that this analysis had minimal heterogeneity and a non-significant trend towards possible better mortality outcomes with doxycycline. Since the highest number of events were recorded in the study by Varghese et al., this study drove the results of the meta-analysis. Mortality was taken as the secondary and not as the primary outcome as the number of outcome events in all treated patients (doxycycline or azithromycin) was anticipated to be low [2]. Doxycycline and azithromycin differ in their side-effect profile, with the former associated with more gastrointestinal side effects and the latter associated with cardiac side effects, such as QT interval prolongation [29]. The meta-analysis indicated that treatment-related adverse effects were similar between the two drugs. This is an important consideration when choosing an antibiotic treatment, as safety and tolerability play a crucial role in patient management. In the study by Varghese et al., neither the doxycycline nor azithromycin arm had any Grade 3 or higher treatment-related adverse event.

It is essential to understand whether the efficacy of the two drugs is comparable or whether one is better than the other. The study results show that the efficacy of both drugs is comparable for clinically relevant outcomes. Historically, doxycycline was considered the drug of choice. Given the current study's findings, azithromycin can be considered a worthy alternative. In clinical settings, individual case-based decisions can be taken based on the dose frequency, availability, cost and patient profile. Although recent studies have shown minimal risk of doxycycline use in pregnancy and children, some clinicians prefer using azithromycin. Doxycycline can be used in patients with old age and myocarditis, where clinicians want to avoid azithromycin due to its effect on QT prolongation [35]. In a retrospective cohort study of older adults with pneumonia, myocardial infarction was higher in azithromycin when compared to other antibiotics [35]. In a study that compared *Clostridium difficile* infection (CDI) between doxycycline and azithromycin, CDI rates were lower with doxycycline [36]. Therefore, doxycycline may be a better choice for individuals with a high risk of CDI. Although doxycycline is the preferred choice for numerous infections involving the central nervous system, some pharmacological studies suggest that azithromycin's blood-brain penetration may be better than doxycycline [37, 38]. Patients who cannot take adequate water or are not able to sit upright are at risk of pill esophagitis with doxycycline [39]. In such cases, azithromycin may be preferred.

In three studies of mild scrub typhus, ≤ 3 days of azithromycin was at least as good as 5-7 days of doxycycline. Patients may prefer the shorter duration and once-daily dosing of azithromycin. In theory, improved adherence to a shorter azithromycin regimen will prevent resistance. Limiting exposure to doxycycline in mild cases might theoretically prevent resistance to doxycycline in other co-existent pathogens such as malaria. A possible approach can be limiting the use of doxycycline to severe cases where there is some evidence supporting its efficacy for scrub typhus. In certain regions where the availability of intravenous doxycycline is limited, advocating for improved access might be beneficial.

It's essential to acknowledge the limitations of this study. Most included studies had a high risk of bias, which could affect the reliability of the findings. Heterogeneity was also observed in the meta-analysis of time to defervescence, possibly due to variations in study populations, dosages, and definitions of outcomes across studies. The doxycycline arm in one of the included RCTs also enrolled patients who received chloramphenicol, which decreased the overall reliability of the doxycycline vs. azithromycin comparison [25]. Most studies did not report mean and standard deviation for time to defervescence. They were calculated using indirect methods from the median, indicating that the primary data might have been skewed from the beginning [9].

## Conclusions

The current data from studies with a high risk of bias did not find statistically significant differences in clinical outcomes between doxycycline and azithromycin for scrub typhus. The choice between these antibiotics should be considered based on availability, cost and patient profile. Further research, including well-designed randomised controlled trials with low risk of bias, is needed to provide more definitive evidence for guiding treatment decisions in scrub typhus.

### Supplementary Information

**Additional file 1:** **Supplementary Table 1.** Databases searched and search string used for the systematic review. **Supplementary Table 2.** Inclusion and Exclusion criteria used for screening and full-text review. **Supplementary Table 3.** Summary of studies with data available for either doxycycline or azithromycin but not both. **Supplementary Table 4.** Additional details of patient selection criteria in terms of the use of diagnostics, and previous antimicrobial use. **Supplementary Table 5.** Fever, mortality, and adverse events related to primary and secondary outcomes in the included studies. **Supplementary Figure 1.** Meta-analysis of doxycycline vs. azithromycin for time to defervescence classified according to age group. **Supplementary Figure 2.** Meta-analysis to calculate the standardised mean difference of time to defervescence between doxycycline and azithromycin with studies stratified according to the severity.**Supplementary Figure 3.** Mean difference of time to defervescence between doxycycline and azithromycin categorised according to whether loading dose was given or not. **Supplementary Figure 4.** Meta-analysis of doxycycline vs azithromycin showing the proportion of patients not achieving defervescence within five days of initiation of drugs. **Supplementary Figure 5.** Meta-analysis of doxycycline vs. azithromycin showing the proportion of patients with treatment-related adverse effects.

## Data Availability

All data generated or analysed during this study are included in this published article [and its supplementary information files].

## Abbreviations

AUFI: Acute undifferentiated febrile illness
RCT: Randomized controlled trial
nRCT: Non-randomized clinical trials
RR: Risk ratio
CI: Confidence Interval
