# Supplementary material for: Doxycycline vs azithromycin in patients with scrub typhus: a systematic review of literature and meta-analysis
Source: BMC Infect Dis. 2023 Dec 18;23:884. doi: 10.1186/s12879-023-08893-7 (PMC10726538; doi:10.1186/s12879-023-08893-7)

Supplementary data

Supplementary Table 1: Databases searched and search string used for the systematic review

Database	Pubmed and Embase
Population	*(scrub typhus[MeSH Terms] OR scrub typhus[Text Word]) OR (orientia tsutsugamushi[MeSH Terms] OR orientia tsutsugamushi[Text Word] OR orientia OR tsutsugamushi) AND (fever[MeSH Terms] OR fever[Text Word]*
Intervention	*(doxycycline[MeSH Terms] OR doxycycline[Text Word]) OR azithromycin[MeSH Terms] OR azithromycin[Text Word])*
Control	

Supplementary Table 2: Inclusion and Exclusion criteria used for screening and full-text review

**Criteria**	**Inclusion**	**Exclusion**
Participant	Individuals of all ages and sexes diagnosed with scrub typhus	• Studies on non-human subjects<br>• Studies focussing exclusively on co-infection
Intervention Comparator	Comparative studies where doxycycline (oral or intravenous) vs azithromycin (oral or intravenous) was used	Non-comparative studies or comparative studies focussing on other drugs
Outcome	Studies where outcome details such as time to defervescence or clinical failure or mortality were reported in each arm	
Study designs	Randomised controlled trials and non-randomised controlled trials (including comparative observational studies)	Case reports, case series, reviews, systematic reviews, conference abstracts and letters to the editor
Settings	All countries	
Language	All languages included	
Publication status	Published up to 20/3/2023	

Supplementary Table 3: Summary of studies with data available for either doxycycline or azithromycin but not both

Sn	Author	Type of Study	Sample size	Diagnostic modality	Drug	Dosage and duration	Average time to defervescence (hours)
1	Song 1995 (10)	RCT	66	IFA	Oral Doxycycline	100 mg BD X 3 days	34 ± 26.5*
2	Watt 2000 (11)	RCT	28	Rapid test or IIP	Oral Doxycycline	100 mg BD X 7 days	52 (4-108)[#]

3	Silpapojakul 2011 (12)	Retrospective cohort study	41	IFA or IIP	Oral doxycycline	Single dose	24 (24-72) [#]
4	Zhao 2016 (13)	Retrospective cohort study	34	ELISA	IV azithromycin	500 mg OD X 5 days	24 (6-126) [#]
5	Lee 2017 (14)	Retrospective cohort study	25	IFA	Oral azithromycin	10mg/kg on D1 f/b 5 mg/kg D2-D5	43.2+3.6*
6	Giri 2018 (15)	Retrospective cohort study	30	ELISA	IV or oral doxycycline	5mg/kg OD	32**
7	Basu 2018 (16)	Prospective cohort study	61	Weil Felix or ELISA	Oral doxycycline	2.2 mg/kg BD X 7-14 days	51.1**
8	Ghosh 2022 (17)	Prospective cohort study	189	ELISA	Oral azithromycin	10 mg/kg single dose	32+22.6*

Abbreviations: RCT- Randomised clinical trial, IFA- Immunofluorescence assay, IIP- Indirect immunoperoxidase assay, ELISA- Enzyme-linked immunosorbent assay *Mean+Standard deviation, **Mean (Standard deviation not mentioned), [#]Median with a range in brackets

Supplementary Table 4: Additional details of patient selection criteria in terms of the use of diagnostics, and previous antimicrobial use

Author	RDT	Weil Felix test	IgM ELISA	IgM IFA	Paired serology	Previous antimicrobial use
Varghese 2023 (18)	Yes					Previous use of >24 hours within 3 days excluded
Guan-Xiu-Gang 2022 (19)		Yes		Yes		
Hwang 2022 (20)				>1:16	4-fold elevation	
Kabir 2022 (21)		>1:80	OD >1			Previous use within 2 weeks excluded
Arun Babu 2021 (22)			OD >0.5			Previous use excluded
Barnabas 2021 (23)			OD >0.5			
Veerappan 2021 (24)	Yes					Previous use excluded
Chanta 2015 (25)	Yes					Previous use within 1 week excluded
Phimda 2007 (26)				>1:400	4-fold elevation	Previous use of >48 hours excluded
Kim 2004 (27)				>1:10	4-fold elevation	

Abbreviations: RDT- Rapid diagnostic test, OD- Optical density value, ELISA- Enzyme-linked immunosorbent assay, IFA-Immunofluorescence assay

Supplementary Table 5: Fever, mortality, and adverse events related to primary and secondary outcomes in the included studies

Sn	Author	Time to defervescence	Clinical failure*	Mortality	Adverse drug effects
1	Varghese 2023 (18)	Secondary	Reported	Secondary	Reported
2	Guan-Xiu-Gang 2022 (19)	Secondary	NR	Primary	NR

3	Hwang 2022 (20)	Primary	Reported	Secondary	NR
4	Kabir 2022 (21)	Secondary	Primary	NR	Reported
5	Arun Babu 2021 (22)	Primary	Primary	NR	NR
6	Barnabas 2021 (23)	Secondary	Primary	Secondary	NR
7	Veerappan 2021 (24)	Secondary	Secondary	NR	NR
8	Chanta 2015 (25)	Secondary	Primary	NR	Reported
9	Phimda 2007 (26)	Secondary	Primary	Reported	Reported
10	Kim 2004 (27)	Primary	Secondary	Reported	Reported

Abbreviations: NR- Not reported
*Clinical failure was defined as failure to attain defervescence in two to three days after initiating the study drugs

Supplementary Figure 1: Meta-analysis of doxycycline vs. azithromycin for time to defervescence classified according to age group

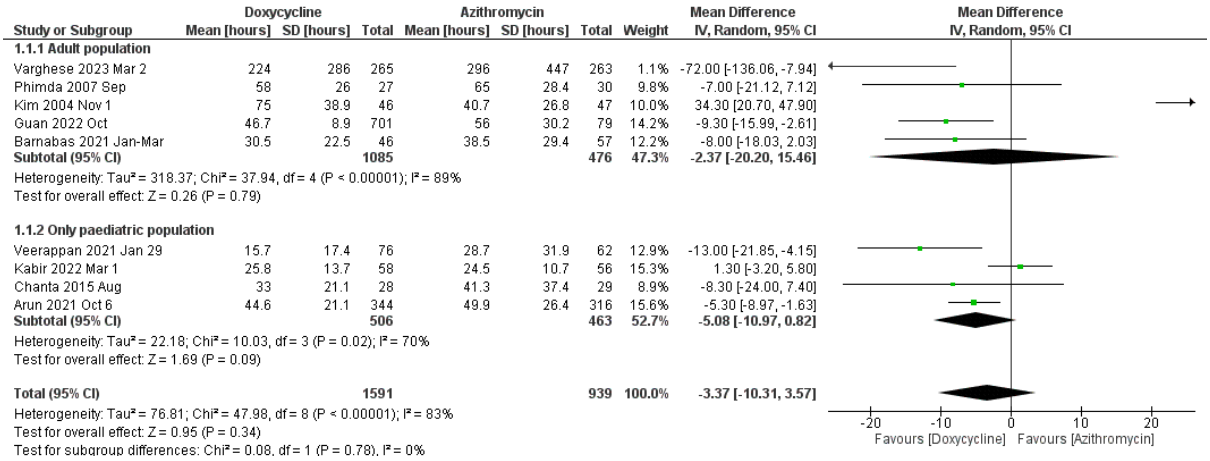

Supplementary Figure 2: Meta-analysis to calculate the standardised mean difference of time to defervescence between doxycycline and azithromycin with studies stratified according to the severity

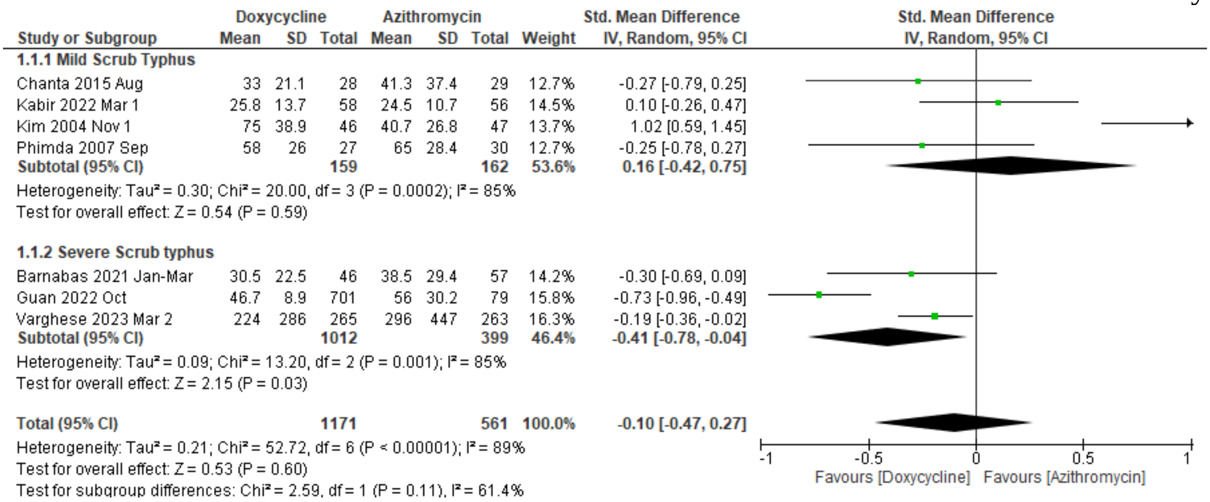

Supplementary Figure 3: Mean difference of time to defervescence between doxycycline and azithromycin categorised according to whether loading dose was given or not

Study or Subgroup	Doxycycline Mean [hours]	Doxycycline SD [hours]	Doxycycline Total	Azithromycin Mean [hours]	Azithromycin SD [hours]	Azithromycin Total	Weight	Mean Difference IV, Random, 95% CI
**1.1.1 Loading dose**								
Chanta 2015 Aug	33	21.1	28	41.3	37.4	29	8.9%	-8.30 [-24.00, 7.40]
Phimda 2007 Sep	58	26	27	65	28.4	30	9.8%	-7.00 [-21.12, 7.12]
Varghese 2023 Mar 2	224	286	265	296	447	263	1.1%	-72.00 [-136.06, -7.94]
**Subtotal (95% CI)**			**320**			**322**	**19.8%**	**-11.62 [-28.30, 5.05]**
Heterogeneity: Tau² = 96.59; Chi² = 3.80, df = 2 (P = 0.15); I² = 47%								
Test for overall effect: Z = 1.37 (P = 0.17)								
**1.1.2 No Loading dose**								
Arun 2021 Oct 6	44.6	21.1	344	49.9	26.4	316	15.6%	-5.30 [-8.97, -1.63]
Barnabas 2021 Jan-Mar	30.5	22.5	46	38.5	29.4	57	12.2%	-8.00 [-18.03, 2.03]
Guan 2022 Oct	46.7	8.9	701	56	30.2	79	14.2%	-9.30 [-15.99, -2.61]
Kabir 2022 Mar 1	25.8	13.7	58	24.5	10.7	56	15.3%	1.30 [-3.20, 5.80]
Kim 2004 Nov 1	75	38.9	46	40.7	26.8	47	10.0%	34.30 [20.70, 47.90]
Veerappan 2021 Jan 29	15.7	17.4	76	28.7	31.9	62	12.9%	-13.00 [-21.85, -4.15]
**Subtotal (95% CI)**			**1271**			**617**	**80.2%**	**-1.41 [-9.29, 6.47]**
Heterogeneity: Tau² = 79.93; Chi² = 43.03, df = 5 (P < 0.00001); I² = 88%								
Test for overall effect: Z = 0.35 (P = 0.73)								
**Total (95% CI)**			**1591**			**939**	**100.0%**	**-3.37 [-10.31, 3.57]**
Heterogeneity: Tau² = 76.81; Chi² = 47.98, df = 8 (P < 0.00001); I² = 83%								
Test for overall effect: Z = 0.95 (P = 0.34)								
Test for subgroup differences: Chi² = 1.18, df = 1 (P = 0.28), I² = 15.2%								

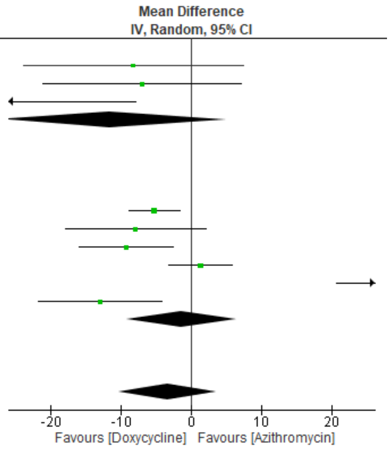

Supplementary Figure 4: Meta-analysis of doxycycline vs azithromycin showing the proportion of patients not achieving defervescence within five days of initiation of drugs

Study or Subgroup	Doxycycline Events	Doxycycline Total	Azithromycin Events	Azithromycin Total	Weight	Risk Ratio M-H, Random, 95% CI
Kim 2004 Nov 1	3	46	0	47	20.4%	7.15 [0.38, 134.66]
Phimda 2007 Sep	0	27	1	30	18.3%	0.37 [0.02, 8.69]
Varghese 2023 Mar 2	8	243	16	225	61.2%	0.46 [0.20, 1.06]
**Total (95% CI)**		**316**		**302**	**100.0%**	**0.78 [0.16, 3.69]**
Total events	11		17			
Heterogeneity: Tau² = 0.85; Chi² = 3.28, df = 2 (P = 0.19); I² = 39%						
Test for overall effect: Z = 0.32 (P = 0.75)						

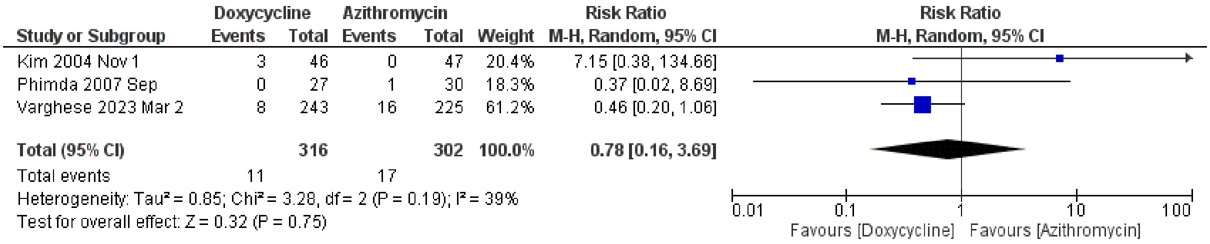

Supplementary Figure 5: Meta-analysis of doxycycline vs. azithromycin showing the proportion of patients with treatment-related adverse effects

Study or Subgroup	Doxycycline Events	Doxycycline Total	Azithromycin Events	Azithromycin Total	Weight	Risk Ratio M-H, Random, 95% CI
Chanta 2015 Aug	0	28	1	29	5.6%	0.34 [0.01, 8.12]
Kabir 2022 Mar 1	5	58	1	56	12.0%	4.83 [0.58, 40.03]
Kim 2004 Nov 1	16	46	13	47	82.4%	1.26 [0.68, 2.31]
**Total (95% CI)**		**132**		**132**	**100.0%**	**1.37 [0.64, 2.94]**
Total events	21		15			
Heterogeneity: Tau² = 0.09; Chi² = 2.20, df = 2 (P = 0.33); I² = 9%						
Test for overall effect: Z = 0.82 (P = 0.41)						

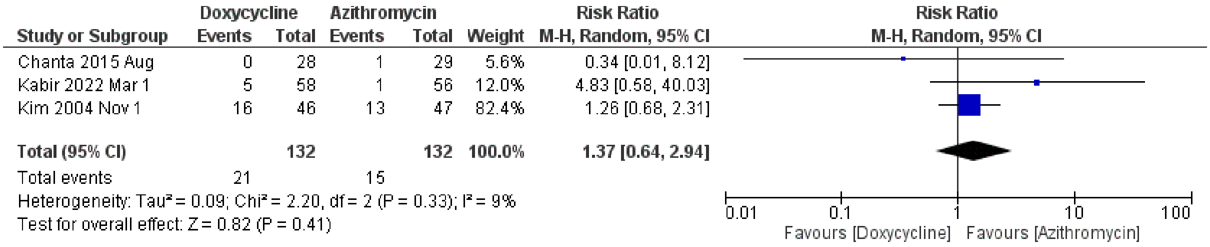

Supplement: Supplementary file 1 — Additional file 1: Supplementary Table 1. Databases searched and search string used for the systematic review. Supplementary Table 2. Inclusion and Exclusion criteria used for screening and full-text review. Supplementary Table 3. Summary of studies with data available for either doxycycline or azithromycin but not both. Supplementary Table 4. Additional details of patient selection criteria in terms of the use of diagnostics, and previous antimicrobial use. Supplementary Table 5. Fever, mortality, and adverse events related to primary and secondary outcomes in the included studies. Supplementary Figure 1. Meta-analysis of doxycycline vs. azithromycin for time to defervescence classified according to age group. Supplementary Figure 2. Meta-analysis to calculate the standardised mean difference of time to defervescence between doxycycline and azithromycin with studies stratified according to the severity.Supplementary Figure 3. Mean difference of time to defervescence between doxycycline and azithromycin categorised according to whether loading dose was given or not. Supplementary Figure 4. Meta-analysis of doxycycline vs azithromycin showing the proportion of patients not achieving defervescence within five days of initiation of drugs. Supplementary Figure 5. Meta-analysis of doxycycline vs. azithromycin showing the proportion of patients with treatment-related adverse effects. [file 12879_2023_8893_MOESM1_ESM.pdf]
